# Immunotherapy in the First-Line Setting in Wild-Type NSCLC

**DOI:** 10.3390/curroncol28060378

**Published:** 2021-11-03

**Authors:** Marie-Hélène Denault, Barbara Melosky

**Affiliations:** 1 BC Cancer, Vancouver Centre, 600 West 10th Avenue, Vancouver, BC V5Z 4E6, Canada; mariehelene.denault@bccancer.bc.ca; 2 Centre de Recherche de l’Institut Universitaire de Cardiologie et de Pneumologie de Québec, 2725 Ch Ste-Foy, Québec, QC G1V 4G5, Canada

**Keywords:** NSCLC, PD-L1, first-line, immunotherapy, non-small cell lung cancer, squamous, nonsquamous

## Abstract

Treatment algorithms in the treatment of advanced non-small cell lung cancer (NSCLC) continue to evolve as new therapeutics show positive efficacy improvements. This review article summarizes the data for the use of immunotherapy for treatment in first-line stage IV NSCLC, organized by the following four sections: single-agent immunotherapy, immunotherapy and chemotherapy, dual immunotherapy, and dual immunotherapy and chemotherapy. The results are summarized and tabulated. Finally, application of the trial data is illustrated in four clinical scenarios depending on the programmed death-ligand 1 (PD-L1) expression levels. Single checkpoint inhibitors have become an easy and excellent treatment in patients whose tumors have high PD-L1 expression. Adding chemotherapy to immunotherapy benefits our patients. Immunotherapy, with or without chemotherapy, is now the standard of care in the first-line setting in patients without EGFR, ALK, or ROS driver mutations.

## 1. Introduction

When we see a newly diagnosed patient with advanced non-small cell lung cancer (NSCLC) as a consult, the first question we ask is, “Does this patient have a driver mutation?”. If not, we start thinking about immunotherapy as an option. We take many factors into account, including performance status, age, comorbidities, and, of course, patient preference. If the patient meets the criteria for treatment, we look to PD-L1 biomarker results to guide the therapeutic choice. Although immunotherapy clinical trials have explored different PD-L1 thresholds, we clinically characterize our patients as PD-L1 ≥ 50%, PD-L1 1–49%, and PD-L1-negative [1]. Other biomarkers we take into consideration include the tumor mutational burden (TMB), and tumor-infiltrating immune cells (IC).

Immunotherapy with checkpoint inhibitors was first introduced into the stage IV NSCLC treatment context in second- and third-line settings, with response rates of approximately 20%, in contrast to the objective response rates (ORR) of 9–13% observed with platinum-based chemotherapy [2,3,4,5]. Most importantly, a subset of patients experienced prolonged survival. Immunotherapy moved, then, into the first-line setting.

Immunotherapy not only includes antibodies designed to target PD-1 (nivolumab pembrolizumab, cemiplimab), and PD-L1 (atezolizumab, durvalumab) biomarkers, but also the cytotoxic T-lymphocyte–associated antigen 4 (CTLA-4), (ipilimumab, tremelimumab).

This review article examines the data for the use of immunotherapy for the first-line treatment of stage IV NSCLC. It is organized by the following four sections: single-agent immunotherapy, immunotherapy and chemotherapy, dual immunotherapy, and dual immunotherapy and chemotherapy. The results are summarized and tabulated. Finally, the practical application of the trial data are illustrated in four clinical scenarios depending on the PD-L1 expression. Fast-moving data in a fast-moving field!

## 2. Single-Agent Immunotherapy

### 2.1. KEYNOTE-024: The Game Changer

Evidence supporting the use of immune checkpoint inhibitors in the first-line setting for metastatic NSCLC first came from the KEYNOTE-024 trial, which compared pembrolizumab to chemotherapy in stage IV treatment-naive NSCLC patients with PD-L1 expressions ≥ 50% [6]. The study population comprised 305 EGFR and ALK wild-type (WT) patients, mostly current or former smokers (92.1%), with nonsquamous histology (81.6%). The patients were randomized to 200 mg of pembrolizumab every three weeks, for up to 35 cycles (two years), versus platinum-based chemotherapy for 4–6 cycles. The trial met its primary outcome, with statistically significant improvements in progression-free survival (PFS) associated with pembrolizumab [6]. The results of the final analyses are now published [7].

Upon progression, 66.0% of patients in the chemotherapy group crossed over to pembrolizumab or another checkpoint inhibitor. The median PFS was 7.7 versus 5.5 months in the pembrolizumab and chemotherapy groups, respectively (Table 1). The overall survival (OS) was analyzed as a secondary outcome and was recently updated in a 5-year analysis, with survival rates of 31.9% versus 16.3%, and a median OS of 26.3 versus 13.4 months [7]. The ORR was higher in the pembrolizumab group (46.1%) than in the chemotherapy group (31.1%).

Treatment-related adverse events (AEs) of any grade were more frequent in the chemotherapy group (90.0%) compared to the pembrolizumab group (76.6%), as were the grade ≥ 3AEs (53.3% versus 31.2%) [7]. Those led to treatment discontinuation in 10.7% versus 13.6% of patients, respectively. Immune-mediated events, on the other hand, occurred more frequently in the pembrolizumab (34.4% any grade, 13.6% grade ≥ 3) than the chemotherapy group (5.3% any grade, 0.7% grade ≥ 3), and were mainly hypothyroidism, hyperthyroidism, and pneumonitis.

By demonstrating the superiority of first-line pembrolizumab compared to platinum-based chemotherapy, KEYNOTE-024 set a new standard of care in metastatic PD-L1-high NSCLC.

### 2.2. IMpower110: Restating the Value of PD-L1

After KEYNOTE-024, strong clinical benefits were supporting upfront single-agent immunotherapy in PD-L1 ≥ 50%. IMpower110 aimed to look not only at PD-L1 expression, but also at the blood-based TMB to better predict the immunotherapy response [8]. We will focus on the former here. A total of 572 stage IV NSCLC patients with PD-L1 ≥ 1% were randomized to atezolizumab, a PD-L1 inhibitor, at 1200 mg every three weeks, versus platinum-based chemotherapy. The EGFR-mutated and ALK-translocated patients could be included provided they had previously received a tyrosine kinase inhibitor (TKI). A protocol amendment was made to exclude EGFR-mutated and ALK-translocated patients (*n* = 18) from the efficacy analysis. A total of 207 (37%) patients had PD-L1 expression levels of ≥50% per the SP142 assay, which was used for patient selection and primary analyses, but this number rose to 260 (49%) and 293 (54%) with the 22C3 and SP263 assays, respectively. This lower sensitivity of the SP142 assay was previously reported in pathology studies [12,13]. The PD-L1 expression was well-balanced between groups, according to the SP142 assay, but the information was not available for the other two assays.

Close to 30% of the patients in the chemotherapy group received other immune checkpoint inhibitors upon progression. The OS was tested hierarchically in the PD-L1-high group (≥50% of tumor cells [TC], or ≥ 10% of ICs), the combined PD-L1-high and PD-L1-intermediate group (≥5% of TC or IC), and, at last, in the whole intention-to-treat (ITT) WT population. The OS was significantly longer with atezolizumab (20.2 months) compared to chemotherapy (13.1 months) (*p* = 0.01) in the PD-L1 ≥ 50% subgroup, while this difference had borderline significance in the high-intermediate group and, thus, could not be tested in the overall population. The OS findings were consistent when using the two other PD-L1 assays. As specified by the protocol’s hierarchical testing strategy, differences in PFS were not formally tested, but seemed to favor atezolizumab in all three groups. The ORR was 38.3% versus 28.6% in the PD-L1-high subgroup, while it was similar between the groups in the high-intermediate group (30.7% vs. 32.1%) and the whole population (29.2% vs. 31.8%).

The rate of treatment-related AEs was 60.5% in the atezolizumab group, compared to 85.2% in the chemotherapy group, for 12.9% versus 44.5% of grade ≥ 3 events, and 6.3% versus 16.3% of events leading to treatment discontinuation. Immune-mediated events occurred more often in the atezolizumab group (40.2% any grade, 6.6% grade ≥ 3) than in the chemotherapy group (16.7% any grade, 1.5% grade ≥ 3), consisting mainly of hepatitis, rash, and hypothyroidism.

In summary, IMpower110 not only restated the benefits of first-line single-agent immunotherapy in the PD-L1 ≥50% subgroup, but also added to the findings of KEYNOTE-024 by demonstrating an absence of benefit in the overall population, driven by PD-L1 1–49% patients. Imbalances in the true proportion of PD-L1-high patients in both groups, created by the lack of sensitivity of the SP142 assay, might have contributed to those findings.

### 2.3. EMPOWER-Lung 1: Is Cemiplimab another Potential First-Line Option?

More recently, the EMPOWER-Lung 1 trial refined the immunotherapy target population by excluding patients with ROS-1 fusions, as well as nonsmokers [9]. However, this trial included patients with stage IIIB and IIIC disease, who were not eligible for chemoradiation. A total of 710 patients with PD-L1 ≥ 50% were randomized to either cemiplimab or a PD-1 inhibitor, at a dose of 350 mg every three weeks for up to 36 cycles (two years), or platinum-based chemotherapy for 4–6 cycles. The majority were stage IV (83.8%) and had nonsquamous histology (55.9%), although, to a lesser degree than seen in other trials for the latter.

Upon progression, 74% of patients in the chemotherapy group switched to cemiplimab, whereas chemotherapy was added for 32% of the patients in the cemiplimab group. The median OS was not reached in the cemiplimab arm and was 14.2 months in the chemotherapy arm (*p* = 0.0002), while the median PFS was 8.2 versus 5.7 months, respectively (*p* < 0.001). The ORR was higher in the cemiplimab group (39%) compared to the chemotherapy group (20%). Increasing PD-L1 expression correlated with larger gains in OS, PFS, and ORR, with the PD-L1 ≥ 90% patients deriving the most benefit.

Treatment-related AEs were more frequent in the chemotherapy group (89% any grade, 37% grade ≥ 3) than in the cemiplimab group (57% any grade, 12% grade ≥ 3). The rates of treatment-related AEs leading to discontinuation were not reported per se, but for treatment-emergent AEs, rates of 4% versus 6% were observed. Immune-mediated events, mainly hypo- or hyperthyroidism and pneumonitis, happened more often in the cemiplimab group (17% versus 2%), but grade ≥ 3 events were seen in only 3%, compared to <1% of patients in the chemotherapy group. Those rates were lower than those seen in previous trials.

In conclusion, EMPOWER-Lung 1, while using a different immune checkpoint inhibitor, confirmed the findings of KEYNOTE-024 and IMpower110, with similar clinical benefits and perhaps less immune-mediated AEs. Although cemiplimab, pembrolizumab, and atezolizumab were not compared head-to-head, the results suggest they are equally effective in the first-line treatment of metastatic PD-L1-high NSCLC.

### 2.4. KEYNOTE-042: The Higher, the Better

Similar to IMpower110, KEYNOTE-042 aimed at extending the role of first-line single-agent immunotherapy to all PD-L1-expressing NSCLC [10]. This study randomized 1274 EGFR and ALK WT, locally advanced or metastatic NSCLC patients with PD-L1 ≥ 1% to either pembrolizumab, 200 mg every three weeks for up to two years, or platinum-based chemotherapy for 4–6 cycles. Three populations were created: PD-L1 ≥ 50% (47.0%); PD-L1 ≥ 20% (64.2%); and PD-L1 ≥ 1%. The distribution was similar between treatment arms. Some characteristics stood out, namely, the high proportion of patients from East Asia (29.0%), and never-smokers (22.1%), but were balanced between populations and arms. The majority (87.4%) of patients had stage IV disease.

Upon progression, 20% of patients in the chemotherapy group received immunotherapy, and 38% of patients in the pembrolizumab group were given chemotherapy. The OS and PFS were both tested hierarchically in the PD-L1 ≥ 50%, PD-L1 ≥ 20%, and, finally, PD-L1 ≥1% patients, with a pre-established alpha boundary that had to be respected to allow further hypothesis testing. In the PD-L1 ≥ 50% population, the median PFS was 7.1 versus 6.4 months (*p* = 0.017) in the pembrolizumab and chemotherapy arms, respectively, compared to 5.4 versus 6.5 months in the PD-L1 ≥1% population. The median OS was 20.0 versus 12.2 months (*p* = 0.0003) for PD-L1 ≥ 50% patients, compared to 16.7 versus 12.1 months for PD-L1 ≥1% patients (*p* = 0.0018). However, the results observed in this last population were driven by the PD-L1 ≥ 50% population, since an exploratory analysis in the 1–49% population did not look significant (13.4 vs. 12.1 months, hazard ratio [HR] 0.92 [95% confidence interval (CI) 0.77–1.11]). OS benefits were consistent across most subgroups, except for never-smokers, with HRs ≥ 1 for all three populations; however, the CIs did not exclude benefit. The ORR was 39% versus 32% in the pembrolizumab and chemotherapy groups, respectively, in the PD-L1 ≥ 50% population, while it was similar between arms in the PD-L1 ≥1% population.

Patients in the chemotherapy arm experienced more treatment-related AEs (90% any grade, 41% grade ≥ 3) than in the pembrolizumab arm (63% any grade, 18% grade ≥ 3), resulting in treatment discontinuation rates of 9% in both arms. On the contrary, AEs of interest, encompassing infusion reactions and immune-mediated events, were more frequent with pembrolizumab than chemotherapy (28% vs. 7% any grade, 8% vs. 1% grade ≥ 3). Pneumonitis, severe skin reactions, and hepatitis were the three most common severe AEs in the pembrolizumab group.

In summary, KEYNOTE-042, although positive in the whole PD-L1 ≥ 1% population, was driven by the PD-L1 ≥ 50% subgroup. This reinforced the superiority of pembrolizumab over chemotherapy in the PD-L1-high population.

### 2.5. CheckMate 026: Setting the Bar too Low

Like IMpower110 and KEYNOTE-042, CheckMate 026 included patients with PD-L1 ≥ 1% but failed to demonstrate significant differences in the PFS or the OS in the primary analysis comparing nivolumab, a PD-1 inhibitor, to chemotherapy in PD-L1 ≥ 5% patients [11]. Of note, randomization was not stratified according to PD-L1 expression, resulting in an unequal distribution of PD-L1 ≥ 50% patients between arms (32% vs. 47%), putting the nivolumab group at a disadvantage. Moreover, there was a high crossover rate from chemotherapy to nivolumab (60%).

Considering the aforementioned studies, the three immune checkpoint inhibitors with proven efficacy for the PD-L1 high population in the first-line setting are pembrolizumab, atezolizumab, and cemiplimab.

## 3. Immunotherapy with Chemotherapy

### 3.1. KEYNOTE-189 and 407: Hitting Harder

While IMpower110, KEYNOTE-042, and CheckMate 026 failed to demonstrate the benefits of first-line single-agent immunotherapy for PD-L1-low patients, KEYNOTE-189 and KEYNOTE-407 hit harder by combining immunotherapy and chemotherapy upfront in this population, for nonsquamous and squamous histology, respectively [14,15]. Both trials looked at the PFS and the OS as coprimary endpoints and were very similar in design. Statistical significance had already been reached in the previously published interim analyses [14,15]. The results of the finalized analyses are presented here [16,17].

In KEYNOTE-189, 616 patients with metastatic WT nonsquamous NSCLC and any level of PD-L1 expression were randomized, in a 2:1 ratio, to pembrolizumab at 200 mg IV or placebo every three weeks for up to two years, both combined with platinum-pemetrexed for four cycles. KEYNOTE-407 included 559 patients of squamous histology, randomized 1:1 to either pembrolizumab-chemotherapy (platinum-paclitaxel or nab-paclitaxel for four cycles) or placebo-chemotherapy. Randomization was stratified according to PD-L1 expression (<1% versus ≥1%) in both trials. The rates of high expression were 32.8% and 26.1% and, hence, the majority had low PD-L1 expression in both trials.

In KEYNOTE-189, crossover from placebo-chemotherapy to single-agent immunotherapy occurred in 55.8% of patients, on and off trial. The PFS was longer in the pembrolizumab-chemotherapy group (9.0 months) than in the placebo-chemotherapy group (4.9 months) (Table 2). A median follow-up of 31 months revealed that the median OS favored the pembrolizumab combination treatment (22.0 versus 10.6 months). The ORR was particularly low in the placebo-chemotherapy group at 19.9%, compared to 48.3% in the pembrolizumab-chemotherapy group, and even rising to 85.7% among those who completed 35 cycles of treatment [17]. In KEYNOTE-407, the crossover rate from placebo-chemotherapy to single-agent pembrolizumab, or another immune checkpoint inhibitor, was 50.5%. The efficacy results were similar to KEYNOTE-189, although more modest. The PFS still favored pembrolizumab-chemotherapy (8.0 months) over placebo-chemotherapy (5.1 months), as did the OS (17.1 vs. 11.6 months) (Table 2). The ORRs were 62.6% and 38.4%, respectively.

A few interesting observations apply to both trials, starting with the PFS findings consistent across all levels of PD-L1 expression and all other subgroups. Incremental PFS improvements were observed with higher PD-L1 expression. Furthermore, looking at the Kaplan–Meier curves for the OS, the absence of early crossing might indicate that the addition of chemotherapy to pembrolizumab mitigates the excessive early deaths observed on single-agent immunotherapy in other trials [8,10]. Of note, OS benefits were seen in PD-L1-negative patients in KEYNOTE-189, but not 407.

Regardless of the relationship to treatment, AEs in KEYNOTE-189 were observed in almost all patients in the pembrolizumab combination (99.8% any grade, 72.1% grade ≥ 3) and the chemotherapy (99.0% any grade, 66.8% grade ≥ 3) groups, leading to treatment discontinuation (any component) in 36.0% versus 17.3% of patients, respectively. As for immune-mediated events, they occurred in 27.2% of the patients in the pembrolizumab combination group, for 12.1% of grade ≥ 3 events. KEYNOTE-407 had a very similar safety profile. Immune-mediated events were a bit more prevalent though, occurring in 35.3% of patients, 13.3% with grade ≥ 3 toxicity. Thyroid disturbances and pneumonitis were the most frequent events, and two patients in each trial experienced grade 5 immune-meditated AEs.

By demonstrating the clinical benefits of pembrolizumab combined with chemotherapy over chemotherapy alone, KEYNOTE-189 and KEYNOTE-407 truly satisfied an unmet need for the PD-L1-low NSCLC population. AEs led to a higher rate of treatment discontinuation in the immunotherapy-chemotherapy combination arm in both trials, emphasizing the importance of patient selection for these potentially more toxic regimens. This has, again, changed the standard of care in metastatic NSCLC.

### 3.2. IMpower130, 131, and 132: Not Enough

Combining atezolizumab and chemotherapy for the first-line treatment of NSCLC, regardless of the PD-L1 expression, IMpower130, 131, and 132, had similar designs and outcomes (Table 2) [18,19,20]. IMpower130 randomized 724 patients with nonsquamous NSCLC to either atezolizumab, 1200 mg IV q for three weeks, with carboplatin-nab-paclitaxel for 4–6 cycles, followed by atezolizumab maintenance, or carboplatin-nab-paclitaxel alone for 4–6 cycles, followed by optional pemetrexed maintenance. IMpower131 focused on patients with squamous histology (*n* = 1021), and added a third arm, carboplatin-paclitaxel, for 4–6 cycles, which was not included in comparisons for the primary analysis. IMpower131 did not include maintenance chemotherapy. Finally, IMpower132, like IMpower130, targeted patients with nonsquamous NSCLC (*n* = 578) and had similar arms, apart from pemetrexed replacing nab-paclitaxel in Impower132. The coprimary endpoints for all three trials were PFS and OS, assessed in the ITT WT population for IMpower130.

The crossover rates from chemotherapy to second-line immunotherapy were high, at 59%, 43.2%, and 45.8% in IMpower 130, 131, and 132, respectively. All three trials demonstrated an increased PFS with atezolizumab-chemotherapy compared to chemotherapy alone (Table 2), and incremental benefits were seen with higher PD-L1 expression in IMpower132. The PFS benefits were seen even in PD-L1-negative patients for IMpower130 and 132. Despite a high crossover rate (59%), a significant increase in OS was observed in IMpower 130 in favor of atezolizumab-chemotherapy (18.6 months) over chemotherapy alone (13.9 months), with borderline statistical significance, however (*p* = 0.033). The OS differences were not significant in IMpower131 and 132. Imbalances in the PD-L1 expression and frequent crossover might account for this negative finding in IMpower132. The ORRs were greater in the atezolizumab-chemotherapy group, compared to the chemotherapy-alone group, in all three trials.

The safety profiles were very similar to KEYNOTE-189 and 407 in terms of severity, cumulative incidence, and discontinuation rate. Rash, hypothyroidism, hepatitis, and pneumonitis were the most frequent events.

Considering these findings, combined atezolizumab-chemotherapy in IMpower 130, 131, and 132 delays progression but does not consistently improve survival in PD-L1-negative and PD-L1-low patients, who are the populations of interest for such a regimen.

### 3.3. IMpower150: Targeting Angiogenesis

On the basis of the potential immunogenic properties of anti-vascular endothelial growth factor (VEGF) inhibitors, IMpower150 investigated the role of the atezolizumab-chemotherapy combination, and the potential added benefits of bevacizumab, in the first-line setting in 1202 patients with non-squamous NSCLC and any level of PD-L1 expression [21,22]. EGFR mutations and ALK translocations were allowed if there had been progression or intolerance to a TKI. Significant vascular disease, bleeding history, recent hemoptysis, and clopidogrel treatment, as well as recently introduced anticoagulants, were bevacizumab-related exclusion criteria. Patients were randomized to either atezolizumab-bevacizumab-carboplatin-paclitaxel (ABCP), atezolizumab-carboplatin-paclitaxel (ACP), or bevacizumab-carboplatin-paclitaxel (BCP). Four to six induction cycles were given every three weeks, and maintenance with atezolizumab and/or bevacizumab, depending on the arm, was then undertaken. Atezolizumab was given at a dose of 1200 mg IV, and bevacizumab at 15 mg/kg.

The crossover rate from BCP to immunotherapy was 46.4%. The coprimary endpoints were the PFS and the OS in the ITT WT population, and in WT patients who had high expressions of an effector T-cell (Teff) gene signature in the tumor, for both the ABCP versus BCP, and the ACP versus BCP comparisons. This analysis will focus on the ITT WT population. The PFS was better with ABCP (8.4 months), but similar between ACP (6.3 months) and BCP (6.8 months) (Table 2). In the publication of the first PFS analysis for ABCP vs. BCP, those findings were consistent across all levels of PD-L1 expression, although more pronounced with higher expression [22]. A significant improvement in OS was also seen in the ABCP vs. BCP comparison (19.5 vs. 14.7 months), although when looking at subgroups, this was significant in the PD-L1-positive, not the PDL-high nor the PD-L1-negative subgroups. By deduction, PD-L1-low patients are the only ones who seemed to survive longer, but this subgroup analysis was not done. No differences in the OS were seen for ACP versus BCP. The ORRs were 63.5% and 48.0% for the ABCP and BCP arms, respectively, but were not reported for ACP.

Secondly, the exploratory analyses of interest in IMpower150 were those of patients with EGFR mutations (*n* = 124) and liver metastases (*n* = 162). For patients with EGFR-sensitizing mutations (exon 19 deletion or Leu858Arg), the ABCP versus BCP comparison suggested improvements in both PFS [10.3 vs. 6.1 months, HR 0.41 (95% CI 0.23–0.75)] and OS [not estimable (NE) vs. 17.5 months, HR 0.31 (95%CI 0.11–0.83)]. The subgroup analysis of patients with liver metastases also implied improved PFS in the ABCP arm (8.2 months) compared to the BCP arm (5.4 months, HR of 0.41 (95% CI 0.26–0.62), and the same was observed for the OS [13.3 vs. 9.4 months, HR of 0.52 (95% CI 0.33–0.82)]).

The toxicity was similar to that reported in KEYNOTE-189 and -407, as well as IMpower130, 131, and 132 for the ACP and BCP arms. However, patients in the ABCP group experienced more grade ≥ 3 treatment-related AEs (60.4%), immune-mediated AEs (48.1%), and AEs leading to the discontinuation of a treatment component (41.2%). Five deaths were caused by pulmonary hemorrhage or hemoptysis, and the majority had high-risk features (tumor infiltration of great vessels or cavitation), leading to increased awareness and surveillance of patients exhibiting those features afterwards.

Putting it all together, IMpower150 is a positive trial, showing longer PFS and OS with the atezolizumab-bevacizumab-chemotherapy combination. Targeting angiogenesis is an important concept, as it is a hallmark of cancer biology. The ABCP regimen may have a niche in EGFR patients who have progressed on prior TKIs, or in those with liver metastases. Of note, EGFR patients have historically derived little to no benefits from immune checkpoint inhibitors.

## 4. Dual Immunotherapy

### 4.1. KEYNOTE-598: When More Is Less

Looking at the favorable data on first-line single-agent immunotherapy in PD-L1-high patients, a question arose as to whether dual immunotherapy would bring additional benefit. KEYNOTE-598 randomized 568 PD-L1 ≥ 50% WT NSCLC patients to pembrolizumab ± ipilimumab, a CTLA-4 inhibitor, for up to two years [23]. ROS-1 fusion was an exclusion criterion, but only in areas where testing and targeted treatments were approved. Participants could stop treatment if they had a complete response (CR) after ≥8 cycles of combined therapy, of which ≥2 were received beyond CR. The study population was similar to that of other trials, and the patient characteristics were well-balanced between groups.

The median PFS and OS were comparable in the pembrolizumab-ipilimumab and pembrolizumab-placebo groups, 8.2 vs. 8.4 months (*p* = 0.72), and 21.4 vs. 21.9 months (*p* = 0.74), respectively (Table 3). These findings were consistent across all subgroups. An ORR of 45.4% was observed in both arms.

Treatment-related AEs occurred in 76.2% of patients in the pembrolizumab-ipilimumab group, compared to 68.3% in the pembrolizumab-placebo group, and in 35.1% versus 19.6% for grade ≥ 3 events. Treatment discontinuation was a consequence in 20.3% versus 37.3% (both agents, or ipilimumab only). Immune-mediated AEs were observed in 44.7% versus 32.4%, respectively, and in no less than 20.2% versus 7.8% for grade ≥ 3 events. Colitis, pneumonitis, and severe skin reactions were the most common severe immune-mediated AEs with combined immunotherapy. Moreover, six patients (2.1%) died of immune-mediated AEs in that group versus none in the pembrolizumab-placebo group. The overall safety profile favored single-agent pembrolizumab.

Therefore, on the basis of the findings of KEYNOTE-598, dual immune checkpoint inhibition with pembrolizumab-ipilimumab brings no additional benefit, and even increases toxicity in the treatment-naive PD-L1-high population, compared to single-agent immunotherapy.

### 4.2. CheckMate 227: The Needle in the Haystack

CheckMate 227 had a complex two-stage design looking at dual immunotherapy, combined immunotherapy-chemotherapy, as well as single-agent immunotherapy, in populations characterized by different levels of PD-L1 expression [24,25]. In this review, we will focus on Part 1A, which randomized 1189 patients with PD-L1 ≥ 1% WT stage IV NSCLC to either nivolumab, 3 mg/kg every two weeks with ipilimumab 1 mg/kg every six weeks, nivolumab, 240 mg every two weeks, or platinum doublet for four cycles [24]. PD-L1 expression was ≥50% in 51.4% of patients, and well-balanced between arms.

The primary analysis compared nivolumab-ipilimumab to chemotherapy. A total of 43% of patients in the chemotherapy group received immunotherapy upon progression, compared to 31.6% of patients in the nivolumab group, who switched to chemotherapy. The primary endpoint for Part 1A was the OS in the PD-L1 ≥ 1% population. The nivolumab-ipilimumab combination was associated with longer OS compared to chemotherapy (17.1 versus 14.9 months, *p* = 0.007) (Table 3), and this was significant across all levels of PD-L1 expression, except for PD-L1 1–49%. In the PD-L1 ≥ 50% patients, the median OS was even longer, 21.2 versus 14.0 months, with an HR of 0.70 (95% CI 0.55–0.90). Although evaluated in a descriptive analysis only, the benefits in PD-L1 < 1% patients seemed significant as well, with an OS of 17.2 versus 12.2 months, favoring nivolumab-ipilimumab. For the other subgroup analyses, no benefits were observed in patients with liver metastases and in never-smokers. Of note, the survival curves crossed at around six months, meaning chemotherapy was still superior early on. PFS was only exploratory in both populations for the nivolumab-ipilimumab versus chemotherapy comparison. In PD-L1 ≥ 1% patients, PFS was 5.1 months in the nivolumab-ipilimumab arm, compared to 5.6 months in the chemotherapy arm. In PD-L1 < 1% patients, it was 5.1 versus 4.7 months. The ORRs were similar between groups: 35.9% versus 30.0% for PD-L1-positive patients, and 27.3% versus 23.1% for PD-L1-negative patients. A four-year update was presented at ASCO 2021, and published results will follow.

Regarding toxicity, the rate of treatment-related AEs was similar between nivolumab-ipilimumab (76.7% any grade, 32.8% grade ≥ 3) and chemotherapy (81.9% any grade, 36.0% grade ≥ 3) groups. However, the AEs in the nivolumab-ipilimumab arm led more often to treatment discontinuation (18.1% vs. 9.1%). The most common potentially immune-mediated AEs were cutaneous (34.0% any grade, 4.2% grade ≥ 3), endocrine (23.8% any grade, 4.2% grade ≥ 3), and gastrointestinal (18.2% any grade, 2.4% grade ≥ 3). Compared to the pembrolizumab-ipilimumab regimen used in KEYNOTE-598, the nivolumab-ipilimumab combination in CheckMate 227 had a similar rate of severe treatment-related AEs but led to treatment discontinuation in fewer cases. The immune toxicity is still more important than in trials using single-agent immunotherapy [6,8,9,10].

In conclusion, CheckMate227 Part 1A demonstrated the superiority of nivolumab-ipilimumab over chemotherapy, in terms of the OS, in the PD-L1 ≥ 1% population.

## 5. Dual immunotherapy with Chemotherapy

### CheckMate 9LA: The Light at the End of the Tunnel

Inspired by the superiority of nivolumab-ipilimumab over chemotherapy in PD-L1-positive patients in CheckMate 227, CheckMate 9LA targeted treatment-naïve WT NSCLC patients, regardless of histology and PD-L1 expression. Most of the 719 randomized patients had nonsquamous histology (69%), as well as PD-L1 ≥ 1% (56.6%), including 24.2% of PD-L1 ≥ 50% patients. The trial’s innovative intervention arm used nivolumab 360 mg IV every three weeks with ipilimumab 1mg/kg IV every six weeks, and two cycles of platinum-based chemotherapy. The comparator was four cycles of platinum-based chemotherapy, representing the standard of care. Immune checkpoint inhibitors could be continued for up to two years, and patients with nonsquamous histology could undergo pemetrexed maintenance.

Of the patients in the chemotherapy group, 37% switched to immunotherapy, and the same percentage in the intervention arm received second-line chemotherapy. After a median follow-up of 9.7 months, the trial met its primary endpoint with increased OS in the nivolumab-ipilimumab-chemotherapy group (14.1 months), compared to the chemotherapy group (10.7 months), with an HR of 0.69 (Table 4). The OS advantage was maintained at a longer median follow-up of 13.2 months (15.6 vs. 10.9 months). Looking at the Kaplan–Meier curves, there is no early crossing, fulfilling the investigators’ objective to mitigate, with two chemotherapy cycles, the excessive early deaths observed in single-agent immunotherapy in other trials [8,10,24,25]. The OS benefit was consistent across all PD-L1 expression subgroups, including the PD-L1-negative patients (16.8 versus 9.8 months, HR 0.62). Patients with squamous histology (HR 0.62) and brain metastases (HR 0.38) were two subgroups of interest, while the survival benefit was negligible for the elderly and never-smoker patients. The PFS (6.7 versus 5.0 months) and the ORR (38.2% versus 25.9%) also favored dual immunotherapy combined with chemotherapy. A two-year update was presented at ASCO 2021, and publication should follow.

The tolerance was comparable to other combination trials, as shown by the treatment-related AE rates of 93.2% in the nivolumab-ipilimumab-chemotherapy group, compared to 88.5% in the chemotherapy group, for grade ≥ 3 rates of 48.9% and 39.5%, respectively. Discontinuation of at least one treatment component ensued in 19% versus 7% of patients. The most common grade ≥ 3 treatment-related AEs with a potential immunological cause in the experimental arm were gastrointestinal (6%), hepatic (4%), and cutaneous (4%).

In summary, CheckMate 9LA is promising in many aspects, first by showing the benefits of a short-lived course of chemotherapy to mitigate the early deaths of immunotherapy, with the possibility of going back to chemotherapy in later treatment lines. It also carries hope for the most challenging subgroups of patients: those with brain metastases, squamous histology, and, last but not least, PD-L1-negative disease.

## 6. How Do You Choose in the Clinic?

Every day, we have to make treatment decisions for our patients with advanced NSCLC. The data presented in this review provides guidance. However, the patients who participate in these large randomized clinical trials are a different population than the usual patients we see in the clinic. Not only are these clinical trial patients heavily prescreened to meet the trial eligibility criteria, but they are more closely monitored during treatment for toxicity and response. Nonetheless, we look to the trials presented and extrapolate to our patients the best we can.

Although we consider the PD-L1 expression levels of our patients when making treatment decisions, we look and think of much more. The treatment that we select for a 40-year-old may be more aggressive than for our patients who are decades older. Patients who have a high burden of disease may need a treatment that promises a rapid response, yet those same patients may have a performance status that precludes a complicated regimen. The patient’s history of autoimmune disease must be carefully explored when an antibody therapy is being considered. The patient’s cognition and ability to recognize side effects, their caregiver support, and even their distance from the treatment center, may affect our choice of regimen and scheduling. Patient preference must also be considered, as some patients refuse, or do not want, chemotherapy. Finally, the patient’s smoking history is always on the back of the mind, as it helps temper both our and the patient’s expectations. Importantly, we must be careful when considering our treatment decisions for patients who are nonsmokers without driver mutations, as they may have a driver mutation yet to be identified. Their response to single immunotherapy may not be ideal.

### 6.1. Patients with PD-L1 >50% NSCLC

The results of KEYNOTE 024 changed the treatment landscape. Single pembrolizumab is usually an easy treatment to deliver, and easy for a patient to receive. As an alternative, the results of IMpower110 imply that, as a PD-L1 inhibitor, atezolizumab may produce a slight reduction in severe immune AEs, as compared to pembrolizumab. The results of EMPOWER-Lung 1 demonstrated that *cemiplimab* may have an advantage at reducing drug wastage, as the dose is single-vial.

In patients who have a high burden of disease, or for whom a rapid response is needed, adding chemotherapy to immunotherapy should be considered. The results from the KEYNOTE 189 and KEYNOTE 407 trials demonstrate that adding chemotherapy to immunotherapy improves efficacy in nonsquamous and squamous advanced NSCLC, respectively.

### 6.2. Patients with PD-L1-Negative NSCLC

For patients whose tumors lack PD-L1 expression, we look to the results of the following trials: KEYNOTE 189 and KEYNOTE 407 with pembrolizumab and chemotherapy, IMpower 150 with atezolizumab and bevacizumab-based chemotherapy, and CheckMate 9LA with a short course of chemotherapy, nivolumab and ipilimumab are all options. Although the results of CheckMate 227 look promising in this setting, this trial was a retrospective study on PD-L1-negative tumors and had an exploratory endpoint.

### 6.3. Patients with PD-L1 1–49% NSCLC

The studies described above that included patients with PD-L1-negative tumors, also included patients whose tumors had PD-L1 levels of 1–49%. Therefore, similar conclusions can be made. Treatments that can be considered for patients with PD-L1 levels of 1–49% include pembrolizumab and chemotherapy (KEYNOTE 189, KEYNOTE 407), atezolizumab and bevacizumab-based chemotherapy (IMpower 150), and a short course of chemotherapy with nivolumab and ipilimumab (CheckMate 9LA).

KEYNOTE 042 demonstrated no benefit in the patient group with PD-L1 expression levels of 1–49% and, although exploratory, clearly the trial results were driven by patients with high PD-L1 expressions. A survival detriment was seen in the single checkpoint inhibitor arm. Similarly, in CheckMate 227, the CI in the patient subset with PD-LI levels of 1–49% crossed unity, indicating a lack of benefit for this group. A recent pooled analysis conducted by the FDA showed the superiority of immunotherapy and chemotherapy over immunotherapy alone in patients with PD-L1 levels of 1–49%, confirming this as the therapy of choice [27].

### 6.4. Caution: Nonsmoker Patients with Wild-Type Tumors

We have learned an important lesson for the treatment of the patient who has never smoked and whose tumor does not have any detectable driver mutations. From all we know about driver mutations, these patients are very unlikely to respond to, or benefit from, single checkpoint inhibitors, even if their tumors express very high PD-L1 levels of ≥50%. The combination of immunotherapy with chemotherapy is needed to treat this patient subset. In KEYNOTE 024, the PFS HR in the never-smokers was 0.90 (0.11–7.59). In IMpower 110, the OS HR with single-agent atezolizumab was 1.83 (0.63–5.31) in the patients who were never-smokers. This is in contrast to KEYNOTE 189, where the OS HR in never-smokers was 0.23 (0.10–0.54). Note that these are all PD-L1 ≥ 50% trials. The important lesson to learn for the treatment of these nonsmoker patients with wild-type tumors is to ignore high PD-L1 expression to determine treatment with single checkmate inhibitors. The best option for the patient who has never smoked, and whose tumor has no driver mutations, is the combination of immunotherapy with chemotherapy.

## Figures and Tables

**Table 1:** Efficacy of single-agent immunotherapy in the first-line setting for advanced NSCLC.

Trial	PD-L1	Arm 1	Arm 2	ORR	PFS	OS
KEYNOTE-024 [6,7]	≥50%	Pembrolizumab	Platinum-based chemotherapy × 4–6 cycles	46.1% vs. 31.1%	7.7 vs. 5.5 months HR 0.50 (95% CI 0.39–0.65)	26.3 vs. 13.4 months HR 0.62 (95% CI 0.48–0.81) 5-year OS 31.9% vs. 16.3%
IMpower110 [8]	≥50%	Atezolizumab	Platinum-based chemotherapy × 4–6 cycles	PD-L1 ≥ 50%: 38.3% vs. 28.6%	PD-L1 ≥ 50%: 8.1 vs. 5.0 months HR 0.63 (95% CI 0.45–0.88)	PD-L1 ≥ 50%: 20.2 vs. 13.1 months HR 0.59 (95% CI 0.40–0.89) *p* = 0.01
				PD-L1 ≥ 5%: 30.7% vs. 32.1%	PD-L1 ≥ 5%: 7.2 vs. 5.5 months HR 0.67 (95% CI 0.52–0.88)	PD-L1 ≥ 5%: 18.2 vs. 14.9. months HR 0.72 (95% CI 0.52–0.99) *p* = 0.04
				PD-L1 ≥ 1%: 29.2% vs. 31.8%	PD-L1 ≥ 1%: 5.7 vs. 5.5 months HR 0.77 (95% CI 0.63–0.94)	PD-L1 ≥ 1%: 17.5 vs. 14.1 months HR 0.83 (95% CI 0.65–1.07)
EMPOWER-lung 1 [9]	≥50%	Cemiplimab	Platinum-based chemotherapy × 4–6 cycles	39% vs. 20%	8.2 vs. 5.7 months HR 0.54 (95% CI 0.43–0.68) *p* < 0.001	NR vs. 14.2 months HR 0.57 (95% CI 0.42–0.77) *p* = 0.0002
KEYNOTE-042 [10]	≥1%	Pembrolizumab	Platinum-based chemotherapy × 4–6 cycles	PD-L1 ≥ 50%: 39% vs. 32%	PD-L1 ≥ 50%: 7.1. vs. 6.4 months HR 0.81 (95% CI 0.67–0.99) *p* = 0.0170	PD-L1 ≥ 50%: 20. vs. 12.2 months HR 0.69 (95% CI 0.56–0.85) *p* = 0.0003
				PD-L1 ≥ 20%: 33% vs. 29%	PD-L1 ≥ 20%: 6.2 vs. 6.6 months HR 0.94 (95% CI 0.80–1.11)	PD-L1 ≥ 20%: 17.7 vs. 13.0 months HR 0.77 (95% CI 0.64–0.92) *p* = 0.0020
				PD-L1 ≥ 1%: 27% vs. 27%	PD-L1 ≥ 1%: 5.4 vs. 6.5 months HR 1.07 (95% CI 0.94–1.21)	PD-L1 ≥ 1%: 16.7 vs. 12.1 months HR 0.81 (95% CI 0.71–0.93) *p* = 0.0018
CheckMate 026 [11]	≥1%	Nivolumab	Platinum-based chemotherapy × 4–6 cycles	26% vs. 33%	4.2 vs. 5.9 months HR 1.15 (95% CI 0.91–1.45) *p* = 0.25	14.4 vs. 13.2 months HR 1.02 (95% CI 0.80–1.30)

All trials described in this table include patients with both squamous and nonsquamous histology. Data are expressed as medians and the intervention arm is mentioned first. ORR = objective response rate, PFS = progression-free survival, OS = overall survival, CI = confidence interval, NR = not reached, NE = not evaluable.

**Table 2:** Efficacy of immunotherapy-chemotherapy in the first-line setting for advanced NSCLC.

Trial	Histology	PD-L1	Arm 1	Arm 2	Arm 3	ORR	PFS	OS
KEYNOTE-189 [14,17]	Non-squamous	Any	Pembrolizumab-platinum-pemetrexed (4 cycles) → pembrolizumab (up to 2 years) → pemetrexed maintenance	Placebo-platinum-pemetrexed (4 cycles) → pemetrexed maintenance	-	48.3% vs. 19.79%	9.0 vs. 4.9 months HR 0.49 (95% CI 0.41–0.59)	22.0 vs. 10.6 months HR 0.56 (95% CI 0.46–0.69) 2-year OS 45.7% vs. 27.3%
KEYNOTE-407 [15,16]	Squamous	Any	Pembrolizumab-platinum-paclitaxel/nab-paclitaxel (4 cycles) → pembrolizumab maintenance	Placebo-platinum-paclitaxel/nab-paclitaxel (4 cycles)	-	62.6% vs. 38.4%	8.0 vs. 5.1 months HR 0.57 (95% CI 0.47–0.69)	17.1 vs. 11.6 months HR 0.71 (95% CI 0.58–0.88) 2-year OS 37.5% vs. 30.6%
IMpower130 [18]	Non-squamous	Any	Atezolizumab- carboplatin-nab-paclitaxel (4–6 cycles) → atezolizumab maintenance	Carboplatin-nab-paclitaxel × 4–6 cycles → optional pemetrexed maintenance	-	49.2% vs. 31.9%	In ITT, WT population: 7.0 vs. 5.5 months HR 0.64 (95% CI 0.54–0.77) *p* < 0.0001	In ITT, WT population: 18.6 vs. 13.9 months HR 0.79 (95% CI 0.64–0.98) *p* = 0.033
IMpower131 [19]	Squamous	Any	Atezolizumab-carboplatin-nab-paclitaxel (4–6 cycles)	Atezolizumab-carboplatin-paclitaxel (4–6 cycles)	Carboplatin-nab-paclitaxel × 4–6 cycles	49.7% vs. 41.0%	6.3 vs. 5.6 months HR 0.71 (95% CI 0.60–0.85) *p* = 0.0001	14.2 vs. 13.5 months HR 0.88 (95% CI 0.73–1.05) *p* = 0.16
IMpower132 [20]	Non-squamous	Any	Atezolizumab-platinum-pemetrexed × 4–6 cycles → atezolizumab-pemetrexed maintenance	Platinum-pemetrexed × 4–6 cycles → pemetrexed maintenance	-	47% vs. 32%	7.7 vs. 5.2 months HR 0.56 (95% CI 0.47–0.67)	17.5 vs. 13.6 months HR 0.86 (95% CI 0.71–1.06) *p* = 0.15
IMpower150 [21,22]	Non-squamous	Any	Atezolizumab-bevacizumab-carboplatin-paclitaxel (4–6 cycles) → atezolizumab-bevacizumab maintenance	Atezolizumab- carboplatin-paclitaxel (4–6 cycles) → atezolizumab maintenance	Bevacizumab-carboplatin-paclitaxel (4–6 cycles) → bevacizumab maintenance	63.5% (ABCP) vs. 48.0% (BCP); not reported for ACP	In ITT, WT population: 8.4 vs. 6.3 vs. 6.8 months ABCP vs. BCP: HR 0.57 (95% CI 0.48–0.67) ACP vs. BCP: HR 0.82 (95% CI0.70–0.97)	In ITT, WT population: 19.5 vs. 19.0 vs. 14.7 months 2-year OS 42% vs. 41% vs. 32% ABCP vs. BCP: HR 0.80 (95% CI 0.67–0.95) ACP vs. BCP: HR 0.84 (95% CI 0.71–1.00) *p* = 0.05

Data are expressed as medians and the intervention arm is mentioned first. Arms colored in gray were not included in the comparisons for the primary outcomes presented here. ORR = objective response rate, PFS = progression-free survival, OS = overall survival, ITT = intention-to-treat, CI = confidence interval, WT = wild type.

**Table 3:** Efficacy of dual immunotherapy in the first-line setting for advanced NSCLC.

Trial	Histology	PD-L1	Arm 1	Arm 2	Arm 3	ORR	PFS	OS
KEYNOTE-598 [23]	Squamous and non-squamous	≥50%	Pembrolizumab- Ipilimumab	Pembrolizumab- Placebo	-	45.4% in both groups	8.2 vs. 8.4 months HR 1.06 (95% CI 0.86–1.30) *p* = 0.72	21.4 vs. 21.9 months HR 1.08 (95% CI 0.85–1.37) *p* = 0.74
CheckMate 227 [24,25]	Squamous and non-squamous	Any	Nivolumab-ipilimumab	Nivolumab	Platinum-based chemotherapy × 4 cycles	PD-L1 ≥ 1%: 35.9% vs. 30.0% PD-L1 < 1% *: 27.3% vs. 23.1%	PD-L1 ≥ 1%: 5.1 vs. 5.6 months HR 0.82 (95% CI 0.69–0.97) PD-L1 < 1% *: 5.1 vs. 4.7 months HR 0.75 (95% CI 0.59–0.96)	PD-L1 ≥ 1%: 17.1 vs. 14.9 months HR 0.79 (97.72% CI 0.65–0.96) *p* = 0.007 PD-L1 < 1% *: 17.2 vs. 12.2 months HR 0.62 (95% CI 0.48–0.78)

Data are expressed as medians and the intervention arm is mentioned first. Arms colored in gray were not included in the comparisons for the primary outcomes presented here. * Prespecified nonprimary analyses. ORR = objective response rate, PFS = progression-free survival, OS = overall survival, CI = confidence interval.

**Table 4:** Efficacy of dual immunotherapy with chemotherapy in the first-line setting for advanced NSCLC.

Trial	PD-L1	Arm 1	Arm 2	ORR	PFS	OS
CheckMate9LA [26]	Any	Nivolumab- ipilimumab- platinum-based chemotherapy (2 cycles)	Platinum-based chemotherapy × 4 cycles → optional pemetrexed maintenance (for nonsquamous)	38.2% vs. 24.9%	6.7 vs. 5.0 months HR 0.70 (97.48% CI 0.57–0.86) *p* = 0.00012	14.1 vs. 10.7 m HR 0.69 (95% CI 0.55–0.87) *p* = 0.00065

Data are expressed as medians and the intervention arm is mentioned first. PD-L1 = programmed death-ligand 1, ORR = objective response rate, PFS = progression-free survival, OS = overall survival, CI = confidence interval.
